# Abstracts and Gleanings

**Published:** 1877-05-20

**Authors:** 


### Abstracts and Gleanings.

The Method of Taxis.—Dr. Dugas, in the New Orleans *Medical and Surgical Journal*, remarks:

The patient should be placed upon a couch of such elevation that the surgeon comfortably seated, may reach the tumor with the right hand without fatigue. The shoulders of the patient should be moderately elevated upon pillows, so as to insure a semi-flexed position of the trunk, while the knees and thighs are also semi-flexed. We have then a complete relaxation of all the muscles interested. The tumor is then grasped with the fingers and thumb in such a manner as to make gentle compression and motion in every direction, for the purpose of driving out of the intestinal noose the grass and liquids it may contain, and thereby reducing the size of the tumor. No violence should be used, and therefore no pain be induced. The surgeon must be well armed with patience, and with the conviction that the reduction can be made neither by violence nor by haste. The patient should be especially directed to be entirely passive, and to avoid any contraction of the abdominal muscles, or expulsory efforts. After fruitless efforts have been continued fifteen minutes the surgeon may need rest, and he may invoke the aid of the patient, inasmuch as most of them have acquired some experience by reducing former decents without professional aid. The patient should then be asked to try his own method while the surgeon is at rest and the result will often be successful. You will find that the patient never gives himself any pain, and usually resorts to gentle motion, back and forth of the tumor. If he fail, a chair should be turned down on his bed in such a manner as to constitute an inclined plane, upon which he should be placed, with his head down, and his pelvis at the highest point. The legs should be flexed, and supported by an assistant. By this position, the gravitation of the abdominal contents will powerfully assist, and sometimes complete the reduction in ten or fifteen minutes. The surgeon should hold up the tumor, move it to and fro, and by pressure from above downward, endeavor to drag the intestines away from the constricted canal. If he fail, let the patient try again.

By repeated and persevering attempts success will most certainly be secured. It is singular that so obvious a procedure as placing the patient upon an inclined plane should not oftener be advised by systematic writers. If any have recommended it, the fact has escaped my observation.

Xanthium Strumarium, or Cockle-bur.—Dr. Kilpatrick, (*in Trans Texas State Medical Association,*) says of the ordinary cockle-bur weed:

"It is a prompt and speedy *vesicant*, and if the leaves are rubbed a little on the skin, there follows a very unpleasant burning sensation, with redness. In order to obtain its vesicant effect, take enough of the leaves, or of the whole plant, mash into a pulp and apply to whatever part you desire to vesicate, and in a short time a good blister will be made.

As an antidote for *snake bite*, you may apply the juice of the plant to the bite and also drink a tea made of the leaves by boiling them in water. Bites of spiders, tarantulas, and centipedes, or any insect, are rendered innocuous by its use in the same way. As a *styptic* to fresh cuts, it is as good as many other of our remedies. To arrest hemorrhage from the uterus, lungs or stomach, the expressed juice applied topically, or drank, acts well and promptly, unless there is a decided hemorrhagic diathesis in the patient. This fact has been announced before this Association on a former occasion, and I have tested it by careful trial.

In all affections of the *kidneys*, or *bladder*; in dysury, strangury, scalding urine, bearing down, and painful micturition, or where there is phosphatic, or other urinary deposit, they are all amenable to its beneficial influence. Take a handful of the leaves, green or cured, and put them in a quart of boiling water and when a decoction is prepared, drink a half cupful, or more, every half hour or hour, until relieved. Generally not more than three draughts are required. I will suggest further, that a tincture of the leaves may be used with the same happy results. In order to secure its full power, the tincture had best be made from the green leaves.

Sub-acute Rheumatic Arthritis.—Dr. J. S. Davis, (in *Medical and Surgical Reporter,*) says of this affection:

The treatment must aim to remove or abate the systemic vice which gives rise to the disease. Gradually, and with much hesitation, I have been led to look upon the iodide of iron as almost a specific for this purpose. The form in which I prefer to use it in these cases is in pills, made according to the formula of the United States Pharmacopœia for 1851, as follows:

R. Ferri sulphat...........dr. j;
Potass. iodid............scr. iv;
Tragacanth pulv........gr. x;
Sacch. pulv.............dr. ss;
Syrup.......................q.s. M.

Ft. mass., et in pil. No. xl div.

Two or three of these pills may be given after each meal. The iodide of potash has not, with me, proved nearly so efficacious in these cases as this preparation, nor have other forms of the iodide of iron. I always have the pills freshly prepared when wanted, and whether it is owing to this fact, or to the presence of sulphate of potash with iodide of iron, resulting from the double decomposition, or to some other cause, I cannot tell, but I have rarely met with a case of subacute rheumatic arthritis which was not arrested by this treatment if used in the early stages.

When the pain is very troublesome the following liniment will often be found to give great relief.

R. Tinct. iod...............
Glycerin.........aa.....oz. j;
Linim. sapon. camph. oz. ij. M.

It is to be borne in mind that nearly every case of this affection is anæmic. Frequently they have the appearance of plethora, but a close observation will almost invariably prove that appearance to be deceptive. This deceptive appearance is generally caused by a fatty deposit, by no means indicative of a healthy state of the blood.

Typhoid Fever, How Produced.—Dr. Simmons, (surgeon to Ken Hospital Yokohama, Japan,) having traced a large number of typhoid cases to parties who used water from the same well in a certain locality, upon investigation elicited the following facts:

1st. That the well in question was fed by a terminal pipe.

2d. That this pipe passed directly through the middle (traversely) of a large sewer.

3. That this pipe was of wood.

4th. That no other well received its supply from this pipe after passing through the sewer.

Taking it for granted that this was the source of the disease, this last fact clearly accounted for the limitation of it to the station, as previous inquiries in the vicinity had failed to discover any cases among those who received their water supply from other wells.

An order was issued to close the well in the station, and open the sewer at the point where the water-pipe crossed it. As was anticipated this was found decayed, and a greater or less exchange of the contents of the two systems going on, and thus was settled beyond a question the source from which the well was contaminated. If any other proof is required as regards this point, we have it in the complete arrest of the disease when this communication was rendered impossible by passing the pipe over the sewer.

Intra-Uterine Injections not Safe.—Dr. Wooten, in report before the Texas Medical Association, says that uterine injections incautiously used are unsafe. He prefers introducing a sponge tent, well prepared, in the evening and removing it next morning, carefully cleansing the parts and applying chromic acid by means of a properly curved probe, wrapped with cotton, and dipped into a solution of the acid of the strength of dr. j. to oz. j. of water. This should be brought in contact with the entire diseased surface, any excess carefully sponged away, and a dressing of cotton soaked in glycerine applied to the parts.

EXTRACT OF LOGWOOD AS A DISINFECTANT.—For twelve years I have used Extract of Logwood as a disinfectant and deoderizer in cancer. I use it in the following manner:—Powdered logwood and hog's lard, of each two ounces. To be mixed and made into a pomade, spread on lint and applied to the sloughing ulcer; the effect is magical, all the odor will disappear in half an hour. The astringency of the logwood will suppress the discharge. No other known agent will fill the indications so well, and yet I have not found a single member of the profession who had any knowledge of the agent until I suggested it. Will some of your numerous readers give it a trial and report the results. —*Drug. Ad.*

SAGE'S CATARRH REMEDY.—According to Adrian Bowers, in the *American Journal of Pharmacy*, this popular nostrum may be reproduced very nearly by employing the following formula and manipulation:

Take of hydrastis canadensis in powder........gr. v.
Indigo .......................gr. ss.
Powdered camphor..... ..gr. ij.
Carbolic acid...............gr. ij.
Sodium Chloride............gr. l.

Rub the camphor with the salt, previously reduced to a moderately fine powder; rub the indigo and carbolic acid together; mix with the salt and camphor, and, lastly, add the hydrastis, and mix intimately in a mortar, without much pressure.

BRIGHT'S DISEASE—NEW DIAGNOSTIC SYMPTOM.—In an article on Bright's disease (in *New York Medical Record,*) a peculiar diagnostic sign is mentioned of the disease, which it is well to note, to-wit: An "unmeaning stare" in the patient's eyes, this with a dullness and sluggishness of the pupil, well dilated, with the presence of moderate œdema of the feet and ankles, persistent headache, occasional attacks of nausea, steady loss of flesh and strength, urine albuminous, with a specific gravity of 1010, constitute an unmistakable array of symptoms of chronic Bright's Disease.

*Treatment:* Make the diet as little nitrogenous as possible. Use milk freely with iron and cod liver oil. The iron may best be given as in the following formula:

R. Tinc. ferri muriatis....
Tinc. nucis vom...aa..gtt. x;
Speritus etheris nitras. dr. j. M.
Give three times a day.

A SECRET REMEDY FOR ASTHMA.—A writer in the *Peninsular Jour. of Med.* says an analysis of "Langell's Asthma Remedy" shows that it is a mixture of ten or twelve parts of coarsely powdered belladonna leaves with one part of nitrate of potassium dried together. The packages contained about two ounces, and were priced at $1.25 each. On igniting a portion on a plate, combustion takes place slowly and the fumes are inhaled. It is said to give prompt relief, is much asked for, and can be sold in quantities for about a half dollar a pound.—*New Remedies.*

BROMHYDRATE OF CICUTINE.—Dr. G H. Boyland, of Baltimore, draws attention to this new principle. It is the alkaloid of the herba cicuta from the conium maculatum. It is strongly alkali, of clear water color in the fresh condition, and soluble in water and alcohol. Dose one one-hundredth to one-fiftieth of a grain. It is not disagreeable in taste, is narcotic in property, bearing the same relation to conium that atropia bears to belladonna.

Treatment of Placenta Prævia.—Dr. R. Davis, of Wilkesbarre, in his address on Obstetrics before the Medical Society of the State of Pennsylvania, in May last advocates the following plan of treatment of placenta prævia, which is a material modification of Barnes' operation: As soon as the os uteri will admit two or three fingers, pass the hand into the vagina. Ascertain by sweeping the finger around between the placenta and uterus (without disturbing their connections) on what side the separation of the placenta is most extensive. That will always be the side of the least extensive attachments. Introduce two or three fingers, on that side, up between the placenta and uterus until the border of the placenta, where the membranes begin, is reached, severing the attachments as you go, if any remain; then hook the fingers over the border and draw the placenta forcibly down and pack it closely to the other side. The membranes will of course, come down with it, and will protrude through the open mouth of the womb. Rupture the membranes at once, and empty the womb of its waters as thoroughly as possible. The head, if it presents, and if pains are active, will now engage in the os, and will crowd the placenta to the side of the cervix, on one side, and will block up the open mouths of the vessels upon the recent seat of the placenta on the other, *and the hemorrhage will cease.* In every case in which I have resorted to this procedure, such has been the happy result, and I have been left free either to allow the labor to end naturally or to end it myself by the forceps.—*Amer. Jour. Med. Science.*

Cold Baths in Infantile Diarrhœa.—Dr. Wocke contributes an article to the *Medizinskoie obosrenie*, in which he refers to the terrible epidemics of diarrhœa which prevail in summer, and which attack with especial severity those infants which are artificially nourished. The epidemic is due in part to the deleterious influence of the elevated temperature on the infantile organism, and in part to the injurious effect which the heat exerts on the aliment, the milk, and the air inspired. To eliminate the first cause, the author recommends cold bathing, from theoretical considerations. The result has been very happy. The wasting children, reduced by vomiting and diarrhœa to a deplorable condition, were as if regenerated by the second day after the baths were commenced. The immovable look and the restlessness disappeared; sleep was restored, the appetite increased, and the diarrhœa diminished. The cold bath acts on the child as a tonic, and enables it to resist the noxious influences, and internal remedies then exert a better influence. Dr. Wocke commences his treatment with cold douches to the head and stomach, then passes to baths, commencing at a temperature of 26° C. and reducing them to 22°. A lower temperature might prove injurious. Three baths a day are sufficient. The author has cured about one hundred cases by this method.—*Lo Sperimentale*, No. 10, 1876.

Deep Injection of Chloroform in Sciatica.—It must be set down as one of the unquestionable evidences of progress in the profession that sciatica, hitherto regarded as among the opprobria medicorum, may be permanently relieved by hypodermic use of chloroform. Dr. L. J. Collins reports an interesting case in Cincinnati *Clinic* of January, relieved by this method. He remarks that, "the pain seemed to originate at a point directly over the sacrô-sciatic foramen, and shooting downward, in the course of the great nerve, to the knee. He seemed to be suffering intensely. After filling my hypodermic syringe with chloroform (about forty minims), I passed the needle its full length into the gluteus muscle, over the point of the emergence of the great nerve from the sacro-sciatic foramen, and injected slowly. He suffered no pain from the injection for some minutes, and then only a dull, aching sensation occurred at the site of injection. In one-half hour his neuralgia had entirely disappeared, and he has had no recurrence since to the present writing. But he called my attention, two days afterward, to a considerable swelling at the site of the chloroform injection; this, however, subsided in three or four days, without any tendency to the formation of an abscess. This I considered a typical case, and one that would well test the merits of chloroform in its treatment. This is the fourth patient upon whom I have used the deep injection of chloroform for the relief of sciatica, and with entire success."

Simple Means to Lessen the Pain of a Blister.—The practice of applying multiple blisters, in acute rheumatism, would everywhere be much more popular with physicians, were it not for the pain, and, in certain cases, the strangury which this mode of treatment produces.

To lessen the one and prevent the other M. Ernest Besnier proposes the following plan: Have care to apply the blisters early in the morning; these properly prepared, covered with a leaf of oiled Joseph paper, will cause very little pain, and never produce the sometimes grave and always painful vesical and renal symptoms which might otherwise occur, provided that the blisters are removed after a few hours, five or six at most, or as soon as the epidermis commences to lift itself lightly and partially, which one can easily tell by the ivory-colored and wrinkled appearance of the skin. It is then time to remove the plaster, which should be replaced by blotting paper, saturated with cerate or cold cream. The vesication then continues almost painless, and the blister is almost as large as if the application of the cantharides had been continued.—J. L. A., *Lyon Medical.*

Piles—Immediate Cure by Igni-Puncture.—Mr. H. A. Reeves, of the London Hospital, has been trying ignipuncture, and found it invariably to rapidly cure piles. He draws down the piles, and then punctures to their bases with conical pointed ends made to fit on to the gas cautery. A dull red heat is required, and two or three punctures suffice for a pile the size of half a walnut. Hard ones to be pierced to their soft attachment. Ulcers and fissures in connection can be touched with the cautery. The bowels are kept confined by a morphine suppository for two or three days. The first motion is painful, but not so bad as before operation, and in a week the patients are discharged cured—a most favorable result which Mr. Reeves contrasts with those obtained by clamps or ligatures.

Treatment of Naso-pharyngeal Catarrh.—In *Pacific Medical Journal*, C. C. Smith, M. D., Cloverdale, Cal., says: "After trying Prof. Thudichum's nasal douche, and several other modes of treatment for naso-pharyngeal catarrh, we finally, some time since, hit upon the idea of using Richardson's apparatus for local anesthesia, to spray a medicated solution into both the nostrils and pharynx. And after trying various astringents, as local medicaments, we have decided, to our own satisfaction, that the sulpho-carbolate of zinc, is as good as any, if not the best. We prepare it thus:

R. Supho Carbolate Zinc .........gr. viij.
Glycerin...................
Water...........aa f oz. ij. M. ft. sol.

Sig: Apply *warm*, once a day thoroughly, for several minutes, to nostrils and pharynx, in strong spray, introducing the spray-tube well up into the nostrils, and deeply into the pharynx.

Hot Water for Treatment of Sick Stomach.—Dr. J. S. Owen in *Medical and Surgical Reporter* says: "I notice in the Reporter several articles on the treatment of sick stomach, and seeing nothing like my own, I thought I would give a few of the many cases in which I have found speedy relief from the use of *hot* water, after the stomach and bowels were emptied, and the patient completely exhausted from vom[illegible]ing and purging

Mrs. W., after th[illegible]sual treatment had failed, and continue[illegible] obstinate heaving for thirty-six hours, at intervals of twenty to thirty minutes, I was called in consultation with her family physician. Found her with cold feet and hands, sunken features, pulse 140 per minute, tongue dry. I ordered half a glassful of *hot* water but she, like all such patients, protested, saying that warm water would only increase the trouble. I remarked [illegible]at I did not intend giving warm water [illegible] hot. In [illegible] few minutes the patient was asleep and had no further trouble. seeing such happy results in a score of cases, I would impress upon those wishing to test it to use water as hot as the patient can bear it.

Spasmodic Asthma.—Dr. Pirtle, in *Medical Brief*, says: I have recently treated several severe cases of spasmodic asthma quite successfully with lumps formed (as many as required) of nitrate potassium and solid ext. stramonium, each lump containing 1 or 2 ounces of each, burnt upon a hot shovel, placed under patient with blanket or sheet thrown overhead. This makes a dense smoke; and of course must be watched, removing the blanket as occasion may require. I also usually prescribe at the same time large doses of bromide and iodide potassium combined in some suitable vehicle. This treatment has given such perfect satisfaction, both to patients and myself, that I thought perhaps it would be worth publishing, not that the remedies are new, but only in the manner of using the nitrate potassa and stramonium.

The Use of Chloroform.—M. Dolbeau, tested the effects, upon persons already plunged in profound natural sleep, of chloroform vapor, a napkin thoroughly saturated being held at the proper distance from the face of the patient, so that he should not be disturbed by contact. The effects were found not to be the same at all ages. In the case of adults the vapor emanating from the napkin generally caused an abrupt awakening with more or less jactitation and sometimes with outcry. With young children the effect was to produce anæsthesia quietly and without arousal. These observations, if fully confirmed, will have a manifestly important bearing on certain questions in legal medicine.—*Exchange*.

NITROUS OXID GAS.—Dr. H. Gibbons (in *Pacific Medical Journal*) thus describes the effects of the inhalation of nitrous oxid gas upon himself:

"I expected to feel its exhilirating effects, which I had many times experienced when inhaling it for amusement. But no such effect was produced; or, if there were, no recollection of it remained. On the contrary, the loss of consciousness was sudden and complete. There was no dreaming nor any sense of the lapse of time. But a sharp, tinkling sound, barely audible, was the only sensation of any kind. In the next moment, I felt a gentle tap on the hand and heard the voice of the operator, and returned in a flash, as it were, to perfect consciousness. I did not realize the lapse of an iota of time from the inhalation of the gas till the restoration of consciousness, and, of course, was much surprised when Dr. Thomas pointed to the table at my side, saying, "There are your three teeth, Doctor!"

He remarks: Nitrous oxid gas is preferable to ether and chloroform as an anæsthetic, because—

1. It is much more easily administered, and is not unpleasant to the patient.

2. It is more speedy and certain in its action, exhibiting its effect in a definite time.

3. Its anæsthesia is as perfect as that from ether and chloroform, if not more so.

4. It has no disagreeable after effects.

5. It is absolutely safe, limiting its paralyzing power to the cerebrum and sensory tract, and not invading the cerebellum or medulla oblongata.

6. It is adapted to all brief surgical operations.

7. Its anesthetic effect may be prolonged by renewal of inhalation, say for five or ten minutes, but should not be prolonged indefinitely. It is therefore not adapted for operations requiring a long time, such as ovariotomy.

COW'S MILK FOR BABIES.—At a meeting the *County of Kings Medical Society* a paper read by Dr. Jerome Walker on "The digestibility and dilution of cow's milk in the artificial feeding of children" gave rise to much discussion. In village and country medical men are not so much troubled in regard to the purity of milk. Yet few things are more perplexing and difficult than the question of how a babe, deprived of its mother's milk, shall be fed. After reading carefully and with much interest the paper alluded to, and the discussion to which it gave rise, we have only to remark that our experience accords with the views expressed by Dr. Mitchell in the following quotation:

"Have tried all kinds of food, with variable results. That which has been most beneficial has been condensed milk, largely diluted with water or barley, gelatine, etc., with lime-water. Milk should be added. There can be no question that when milk has once become cold it is never again the same. It is impossible to foretell on what a child will thrive—sometimes it will be sugar pure and simple; sometimes clear butter; and after that some one of the prepared foods. Borden's condensed milk has always been good."

Quite a variety of recipes and methods of preparation were suggested by members. One, mentioned by Dr. Chapman, struck us with favor, to-wit:

| | | |
|---|---|---|
| Condensed Milk, | 2 | teaspoonfuls. |
| Lime-water, | 4 | " |
| Sugar, | ½ | " |
| Water tepid, | 24 | " |

Mix.

TRACHEOTOMY WITH THE THERMO-CAUTERY.—*L' Union Medicale du Canada* quotes from the *Bordeaux Med. Gazette* the description of the operation of tracheotomy as performed by the galvanic cautery. The instructions are to provide a platina knife properly heated and proceed thus:

1. Cut slowly from above downwards in the median line, immediately above the edge of the cricoid cartilage, and, with a single incision, the skin and superficial aponeurosis.

2. Starting from the same point, divide slowly and with one cut the muscular tissues, down to the trachea.

3. Apply the point of the knife a third time to the superior extremity of the incision, and thrust it in perpendicularly until the sensation of resistance is overcome. The characteristic sound of the entrance of air will be heard. Proceed at once to enlarge the incision. This part of the process must be done quickly, in order to avoid injuring the surrounding parts by the heat of the instrument. The operation was successfully performed in three cases. It requires less than a minute to make all three incisions. Before opening the larynx it may be fixed against the surrounding parts by pressure with the thumb and fingers of the left hand. This operation requires but a short time, it is entirely free from hemorrhage, and it may be performed by one person, no assistance being required except to hold the child.

CHROMIC ACID IN THE TREATMENT OF ULCERATING GRANULATIONS OF THE OS UTERI.—In the *Annales de la Societe de Medicine de Gand*, M. Kœberle prefers chromic acid as a cauterizing agent to the other remedies usually used, as pernitrate of mercury, iodine, nitrate of silver, and the actual cautery. He uses it in the crystalloid condition. It is a very anhydrous substance, and readily absorbs the moisture from the tissues which it may touch. M. Kœberle applies it through an India-rubber speculum on a tampon of cotton-wool. Vomiting often supervenes within fifteen or twenty minutes from the application of the acid. When the tissues are seriously altered it is necessary to repeat the cauterization, but M. Kœberle has hitherto found three applications to suffice. After the application he applies a tampon, and advises the patient to use two soap-and water injections daily. He treats all ulcerations of the os in this way, as in epithelioma.—*London Med. Rec.*, March 15, 1877.

CANDERNA ON THE TREATMENT OF ACUTE INTESTINAL CATARRH IN INFANTS AND SUCKLINGS.—The author remarks (*Med-Chir. Centralblatt*, No. 51, 1876), that the common cause of this affection is injudicious or excessive feeding. The first indication, therefore, is to get rid of the offending matter. For this purpose castor-oil, mixed or not with aqueous extract of rhubarb, is the best. The excessive peristalic action of the intestines is diminished by the application of an opiative poultice. The second indication, the removal of the catarrh set up by the irritating substance is best accomplished with aqua laurocerasi, given alternately with minute doses of calomel every two hours.—*London Medical Rec.*, March 15, 1877.

TREATMENT OF VESICAL HEMMORRHAGE.—The general treatment must be based upon the cause of the hemorrhage, but the bleeding itself should be checked by the application of an ice-bag over the bladder and perineum, the internal administration of astringents (alum tannin), or the injection, as advised by Lebert of a 3-5 per cent. solution of tannin into the bladder. Lallemand has cauterized the bladder. In case of the formation of coagula, the bladder must be emptied by means of the catheter. KRAUS.—*Diagnose und Therapie der Krankheiten des Menschen.*

RHUS-POISONING.—Dr. F. L. James, of Arkansas, says in *New Remedies*, that having discovered that the serum which exudes from the pustules in rhus-poisoning was intensely acid, concluded to try the bicarbonate of soda, and found it very efficacious. Next to the soda bi. carb. the following remedies have afforded the best results in my hands, in the order named: borax dissolved in glycerine, (boracis, dr. i; glycerinæ, dr. i); aqua ammonia (ammon. aquæ. fl. dr. ij; ol. oliv., fl. ox. i); sugar of lead (a wash of the saturated solution or the liquor plumb. diacet. diluted). Quinine and iron, administered internally in those coses where a tonic was needed, hastened the cure."—*Louisville Med. News.*

ELECTRICITY IN HERPES ZOSTER, CANCER, ETC.—Dr. A. D. Rockwell, (*New York Medical Journal*) says: "In the treatment of the pain of herpes zoster, galvanism is invaluable. In many cases that have fallen under my observation, I have never known it to fail to afford either complete or approximate relief. The effects of galvanism on the extreme suffering that so often accompanies mammary cancer are often little short of magical. I have in many instances seen the acutest agony relieved instantly, and, while this relief is necessarily seldom if ever permanent, it is possible in many cases, by repeated applications, to keep the pain in abeyance for months.

BACTERIA—THEIR NATURE.—Dr. T. E. Satterthwaite (*Med. Record, Dec.*, 1875,) says: "1. Bacteria are certain vegetable organisms which probably belong to the algæ; they are found abundantly in nature, but chiefly where there is moisture. 2. They exist in the body in health covering the mucous membranes from the mouth to the arms, and sometimes to penetrate a certain distance into the system without causing symptoms of disease. 3. They also exist in putrifying fluids and in various diseased processes, occurring in hot and cold abscesses, in the blebs of erysipelas and in simple blisters."

BROMHYDRATE OF CONIA.—The salt has been used by various practitioners with great success in the treatment of whooping-cough, in doses of about one-twelfth of a grain, if necessary, every hour, for a child three years of age; or one-thirtieth of a grain for a child of one year; or one-sixth of a grain for adults. In sciatica it has been employed hypodermically in quantities of one-twelfth of a grain with good results.

AN ELECTUARY AGAINST TAPEWORM.—Dr. Du Plessis gives the following prescription:—Kameela, 6 to 12 grammes; pulp of tamarinds, 30 to 40 grammes; cedar juice and syr. cort. aurant. [illegible] *ib.* It is taken in one dose in the morning before breakfast. With bothriocephalus the cure is generally a radical one with one dose. For tænia a stronger dose is advisable.—*Med. Times.*

CURABILITY OF SYPHILIS.—Prof. Zeissl in a clinical lecture on the subject of the curability of syphilis, said: "I tell you, gentleman, that if a man contract syphilis he will die syphilitic, and at the day of judgment his ghost will have syphilis."

